# Clinical Recognition of Sensory Ataxia and Cerebellar Ataxia

**DOI:** 10.3389/fnhum.2021.639871

**Published:** 2021-04-01

**Authors:** Qing Zhang, Xihui Zhou, Yajun Li, Xiaodong Yang, Qammer H. Abbasi

**Affiliations:** ^1^First Affiliated Hospital of Xi’an Jiaotong University, Xi’an Jiaotong University Health Science Center, Xi’an Jiaotong University, Xi’an, China; ^2^Northwest Women’s and Children’s Hospital, Xi’an Jiaotong University Health Science Center, Xi’an, China; ^3^School of Electronic Engineering, Xidian University, Xi’an, China; ^4^James Watt School of Engineering, University of Glasgow, Glasgow, United Kingdom

**Keywords:** cerebellar ataxia, clinical recognition, microwave, sensory ataxia, wireless sensing technology

## Abstract

Ataxia is a kind of external characteristics when the human body has poor coordination and balance disorder, it often indicates diseases in certain parts of the body. Many internal factors may causing ataxia; currently, observed external characteristics, combined with Doctor’s personal clinical experience play main roles in diagnosing ataxia. In this situation, different kinds of diseases may be confused, leading to the delay in treatment and recovery. Modern high precision medical instruments would provide better accuracy but the economic cost is a non-negligible factor. In this paper, novel non-contact sensing technique is used to detect and distinguish sensory ataxia and cerebellar ataxia. Firstly, Romberg’s test and gait analysis data are collected by the microwave sensing platform; then, after some preprocessing, some machine learning approaches have been applied to train the models. For Romberg’s test, time domain features are considered, the accuracy of all the three algorithms are higher than 96%; for gait detection, Principal Component Analysis (PCA) is used for dimensionality reduction, and the accuracies of Back Propagation (BP) neural Network, Support Vector Machine (SVM), and Random Forest (RF) are 97.8, 98.9, and 91.1%, respectively.

## Introduction

“Ataxia” was initially used to describe various uncoordinated characteristics of different diseases, such as gait, movement, heartbeat, etc. Now it is more specifically used to express the symptoms of motor mismatching synchronization and balance disorder after the brain, cerebellum, deep sensation (proprioception), vestibular and other systems are damaged (Bastian, 1997). Different pathological locations often show different characteristics. Sensory ataxia is caused by the impairment of somatosensory nerve, which leads to the interruption of sensory feedback signals and therefore, the body incoordination is caused. For Cerebellar Ataxia patients, the Romberg’s sign was positive, the typical symptoms include walking slowly, rolling, etc. Symptoms were mild when eyes were open and aggravated when eyes were closed (Fadic et al., 1997; Donnelly, 2011). Cerebellar ataxia patients are more common, it is a loss of body muscle coordination caused by cerebellar disease. Trunk ataxia often indicates cerebellar vermis lesions, and limb ataxia often indicates cerebellar hemisphere lesions. The corresponding patients often have eye tremor, low muscle tension, unclear speech, and other symptoms (Diener and Dichgans, 1992; Bastian et al., 1996).

In clinical testing, SA syndrome is very easy to be misdiagnosed as CA syndrome, which leads to the inability of patients with ataxia to get correct diagnosis and treatment in time. At present, several international medical organizations have formed to study ataxia (Klockgether and Paulson, 2011), and several ataxia assessment scales were developed, such as “International Cooperative Ataxia Rating Scale for pharmacological assessment of the cerebella syndrome (ICARS)” (Trouillas et al., 1997), “Scale for the assessment and rating of ataxia (SARA)” (Schmitz-Hübsch, 2006). Some scholars have also done relevant research on the clinical detection and differentiation of SA and CA symptoms, and given the clinical diagnosis method (Chhetri et al., 2014). Both the assessment scale and related research work have referred to two basic indicators: Romberg’s sign and gait; which could be used for Clinical detection and differentiation of SA and CA.

### Romberg’s Sign

The maintenance of human balance mainly depends on the coordination of vestibular system, visual system and proprioceptive system (Maurer et al., 2001). In an upright position, a normal person can stand steadily when the eyes open and close; but when two or more systems are damaged, the human body will not be able to maintain balance. For example, when a patient is suffering from Sensory Ataxia, the visual system can provide compensation information when the eyes are open, so the patient can remain upright and stable; Visual compensation would disappear when the eyes are closed, patients will not be able to maintain upright stability. This is the theoretical basis of Romberg’s sign has become an important part of modern neurological clinical examination (Lanska, 2002).

**FIGURE 1:**
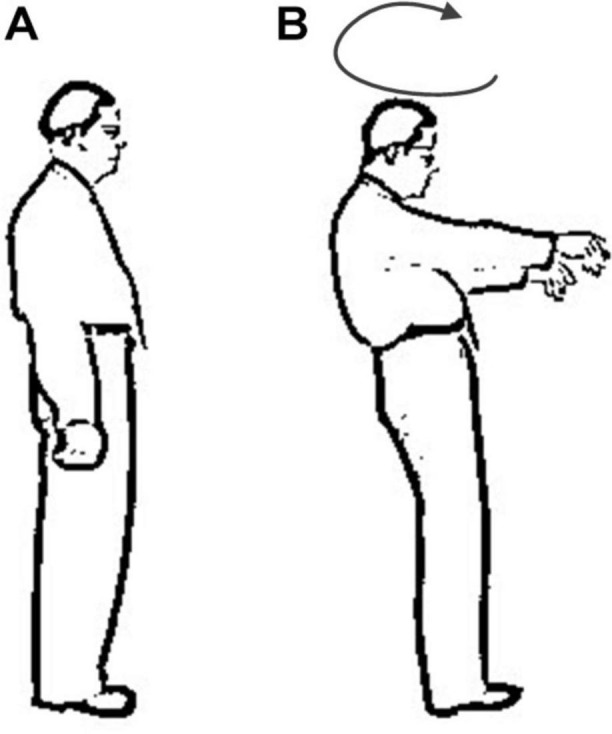
Comparison of different performance of patients with sensory ataxia in Romberg’s test.

**FIGURE 2:**
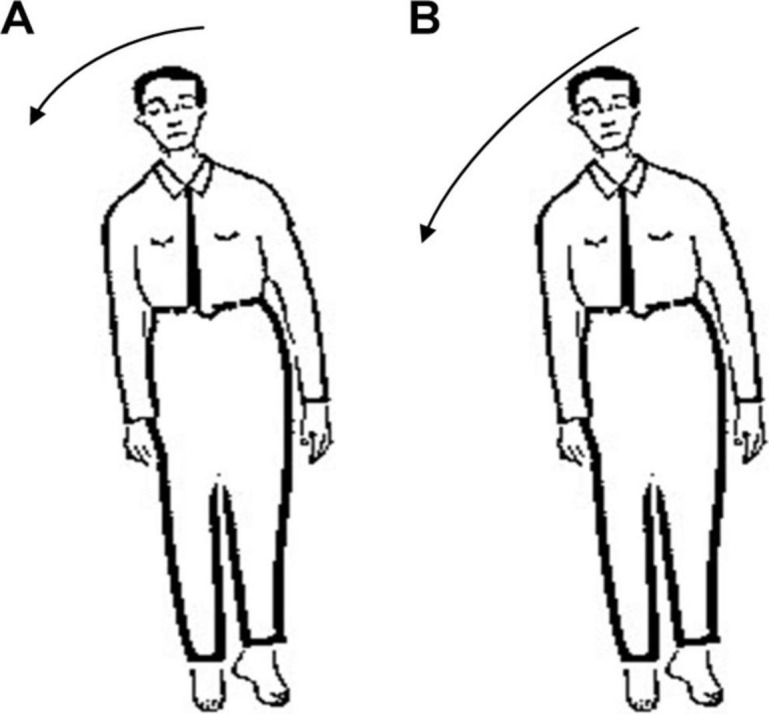
Comparison of different performance of patients with cerebellar ataxia in Romberg’s test. **(A)** Eye opening stage and **(B)** eye closure stage.

In Romberg’s test, the patient’s feet are closed and arms are placed on both sides of the body. Standing is divided into two stages: opening eyes and closing eyes. Firstly, the patients are allowed to open their eyes and stand for a certain time, then the patients close the eyes and stand for a while, and the patients are observed: whether their body have obvious shaking in two stages. As long as there is a stage in which the patient shows standing instability, the Romberg’s sign is positive (Pearce, 2005). Before carrying out Romberg’s test, lower limb diseases or other factors should be excluded. In order to prevent the patient from falling down, protective pads should be laid around the patient’s standing and medical staff should also take care of the patients. During the experiment, normal people can keep their body stable whether they open or close their eyes. Considering age, gender and other factors, the normal performance of the minimum standard should be that body balance can be maintained for 6 s during eye closure (Hain and Cherchi, 2017). For sensory ataxia and cerebellar ataxia, their Romberg’s signs are both positive, but there are some differences. The patient can keep standing steady during the eye-opening phase, and standing unsteadily, wobbling, or even falling in the closed eye phase (Franchignoni et al., 1984), as shown in Figure 1. The cerebellar ataxia patients were unstable in the stage of closing eyes and opening eyes, and tend to tilt toward the diseased side of cerebellum (Cazzato et al., 2016), as shown in Figure 2. Romberg’s test is a simple and sensitive clinical trial, the different performances of normal people, sensory ataxia patients and cerebellar ataxia patients in the Romberg’s test are given in Table 1.

**TABLE 1:** Performance of different groups in Romberg’s test.

**Groups**	**Open eyes stage**	**Close eyes stage**
Normal	Stable	Stable
Sensory ataxia	Stable	Instable
Cerebellar ataxia	Instable	Instable

### Gait Detection

Abnormal gait can be caused by motor or sensory disturbance, and its characteristics are related to the location of lesion. It can be seen in many diseases in nervous and other systems; some typical abnormal gaits have implications for certain diseases (Thomann and Dul, 1996).

#### Sensory Ataxia Gait

**FIGURE 3:**
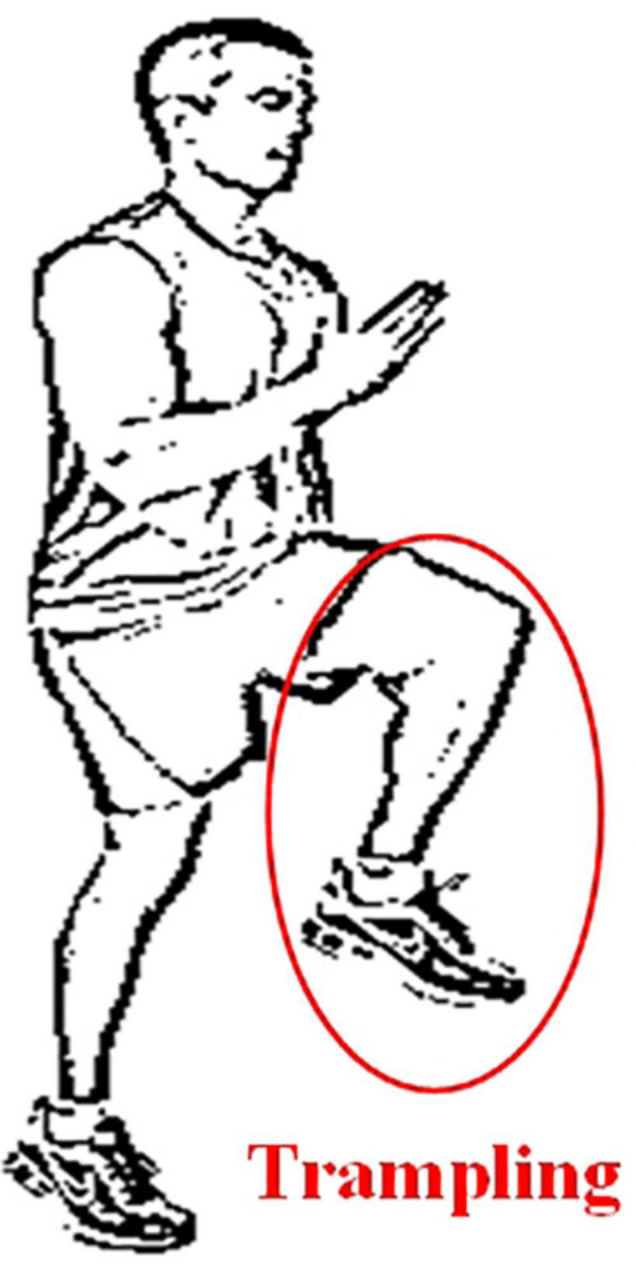
Sensory ataxia Gait.

When a normal person walks, the sensory nerve would be stimulated when the sole of the foot touches the ground, then the relevant information is transmitted to indicate the position of the feet. Since the patients with sensory ataxia lose the input of the stimulus, in order to know the time and place the feet land, the patient would put his feet on the ground heavily. The key to this gait is that when patients can’t see their feet (e.g., in the dark), stepping will increase obviously. This gait is sometimes referred to as stepping gait, because patients may lift their legs to a very high position (Missaoui et al., 2013). The sensory ataxia gait diagram is shown in Figure 3.

#### Cerebellar Ataxia Gait

**FIGURE 4:**
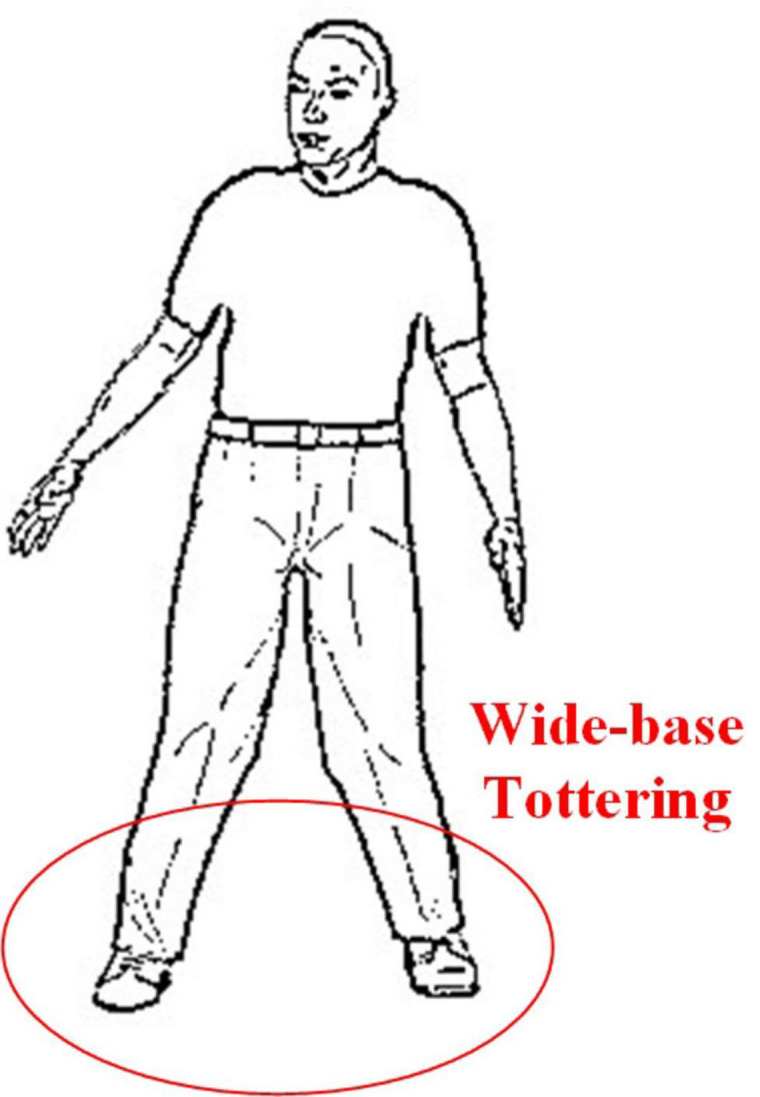
Cerebellar ataxia Gait.

This gait is common in cerebellar diseases and is often described as a clumsy, tottering, and wide-base gait. Similar to the gait after acute alcoholism, patients will not be able to walk straight. Patients with greater trunk instability during walking are more likely to have lesions in the midline vermis of the cerebellum (Mochizuki and Ugawa, 2010). The cerebellar ataxia gait diagram is shown in Figure 4.

At present, there are many related works on quantifying the degree of swing in Romberg’s test.

One of the common ways is wearing facilities such as pressure sensor, gravity acceleration sensor, etc. (Diener et al., 1984; Lanska, 2002; Mcgough et al., 2018); other ways include collecting videos via cameras (Havasi et al., 2007), etc. Currently, many related works have been done for Romberg’s test and gait detection purpose. Pressure sensors, gravity acceleration sensors, videos, and some other approaches have been applied in this domain (Diener et al., 1984; Lanska, 2002; Zongyi and Sarkar, 2006; Havasi et al., 2007; Afendi et al., 2013; Umair Bin Altaf et al., 2015; Wang et al., 2017; Mcgough et al., 2018). The methods in previous work have their respective advantages; however, some issues like self-consciousness enhancing, abnormal mood changes cannot be ignored. Non-contact wireless sensing technology could avoid these problems and by using omnidirectional antennas, Romberg’s test and gait detection can be achieved.

The steps can be summarized as follows: firstly, the microwave sensing system working at 4.8GHz was used to collect original perception data; then, the data were preprocessed; finally, the features are extracted and three machine learning algorithms [Back Propagation (BP) Neural Network (Rumelhart et al., 1995), Support Vector Machine (SVM) (Cortes and Vapnik, 1995) and Random Forest (RF) (Shi and Horvath, 2006)] were applied to train the models. The experimental results show that the accuracies of three algorithms are higher than 96% for Roberg’s test and gait detection, demonstrating the feasibility and effectiveness of the method.

The contribution of this paper can be summarized as follows: (1) detection and distinguishing of sensory ataxia and cerebellar ataxia can be achieved by using wireless sensing technology, and the patients’ privacy can be protected; (2) Romberg’s test and gait detection are validated, thus the accuracy of clinical diagnosis can be improved; (3) various machine learning algorithms are used to increase the stability and credibility of the results.

The rest of the paper is organized as follows: the principle of wireless sensing is introduced in part II; in part III, the experimental devices and scheme are described in detail; in part IV, the data for Romberg’s test and gait detection are analyzed; and the experimental results are discussed in part V; and in part VI, the full paper is summarized.

## Principle of C-Band Wireless Sensory

In typical indoor environment, the wireless signal emitted by the transmitter would be affected by the objects or the human body; and the refraction, reflection and diffraction may cause multipath effect. These homologous wireless signals in different propagation paths show different physical characteristics at the receiving end, such as the amplitude and phase of the receiving signals, which contain rich information from the external environment.

When the receiver detects that the signal changes, it indicates that the external environment has been changed. By de-noising the acquired data and further processing with classification algorithm, we can reduce the environmental factors that lead to the change of the received signal, so as to obtain the desirable information in the environment. In this work, since the antennas were used for detection and monitoring applications in a regular shape room, basic omnidirectional monopole antennas were considered; for irregular shape space and room, specially designed antennas would be necessary to enhance the performance and accuracy of sensing. The main differences between received signal strength and channel state information are explained in Zhu and Zhang (2010) and Zheng et al. (2013). CSI considers the number of antennas and subcarriers, and can measure more fine-grained information, the facility which confirms to the IEEE 802.11n standard was used to collect the CSI data. The IEEE 802.11n standard uses orthogonal frequency division multiplexing (OFDM) to transmit a single data stream with 20 MHz bandwidth through 56 orthogonal subcarriers, the signals transmitted on each subcarrier have different signal strength and phase (Lorincz and Begusic, 2006). The facility used in this paper provides 30 available subcarriers to users. Next, we will further explain the principle of C-Band wireless sensing measurement from the formula.

It is known that the channel impulse response (CIR) is generally used to describe the multipath effect in wireless channels. Under linear time-invariant conditions, the CIR can be expressed as follows,

(1) $$\text{h}\left(\tau \right)=\sum_{i=1}^{N}a_{i}e^{-j\,\theta_{i}}\delta \left(\tau -\tau_{i}\right)\left(i=1,2,\ldots ,N\right)$$

In the formulas above, *a*_i_, θ_i_ and τ_i_ represent the attenuation factor, phase shift, and time delay of the i-th path, respectively, N is the total number of propagation paths, and δ(τ) is Dirichlet pulse function.

Since the multipath propagation of signals can cause delay and attenuation, we can also describe the channel by channel frequency response (CFR), as shown in (2),

(2) Y=H⁢X+N⁢(2)

Where Y is the vector representation of receiving signal, X is the vector representation of transmitting signal, N is the noise matrix, H is the channel attenuation matrix and describes the attenuation factor of signal on each transmission path, the dimension of H can be expressed as:

(3) $$D\,i\,m_{H}=R_{N}\times T_{N}\times S\,u\,b_{N}$$

Where R_*N*_ and T_*N*_ are the number of receiving antennas and transmitting antennas, respectively.Sub_*N*_ is the number of subcarriers.

CSI is essentially a representation of the frequency response of each subcarrier channel, as shown in (4),

(4) $$\text{h}\left(f_{i},t\right)=|\begin{array}{c} \text{h}\,\left(f_{i}\,,t\right) \end{array}|\times \arg\left(h\left(f_{i},t\right)\right)\left(\text{i}=1,2,\ldots ,30\right)$$

In (4),|h(*f*_*i*_, t)|, arg(h(*f*_*i*_, t)), and*f*_*i*_ denote the amplitude, phase, and central frequency of i-th subcarrier, respectively.

Since the patient takes some time to perform Romberg’s test and gait detection, we need continuous monitoring, and the received CSI data can be expressed as:

(5) $$\text{D}=\left[P_{1},P_{2},\ldots ,P_{n}\right]$$

Where D represents the data stream received by the receiving antenna, P_*i*_ (i = 1, 2…, n) represents packet. Each packet contains 30 subcarriers, and n is the total number of received packets. D constitutes the analysis data source for detecting and distinguishing sensory ataxia and cerebellar ataxia. Since the phase of subcarriers in each packet is random, this paper will mainly use the amplitude information of subcarriers (Yang et al., 2017).

## The Experiment Design

The experiment was carried out in an approximate ward room, its size is 7 m×5 m.

**FIGURE 5:**
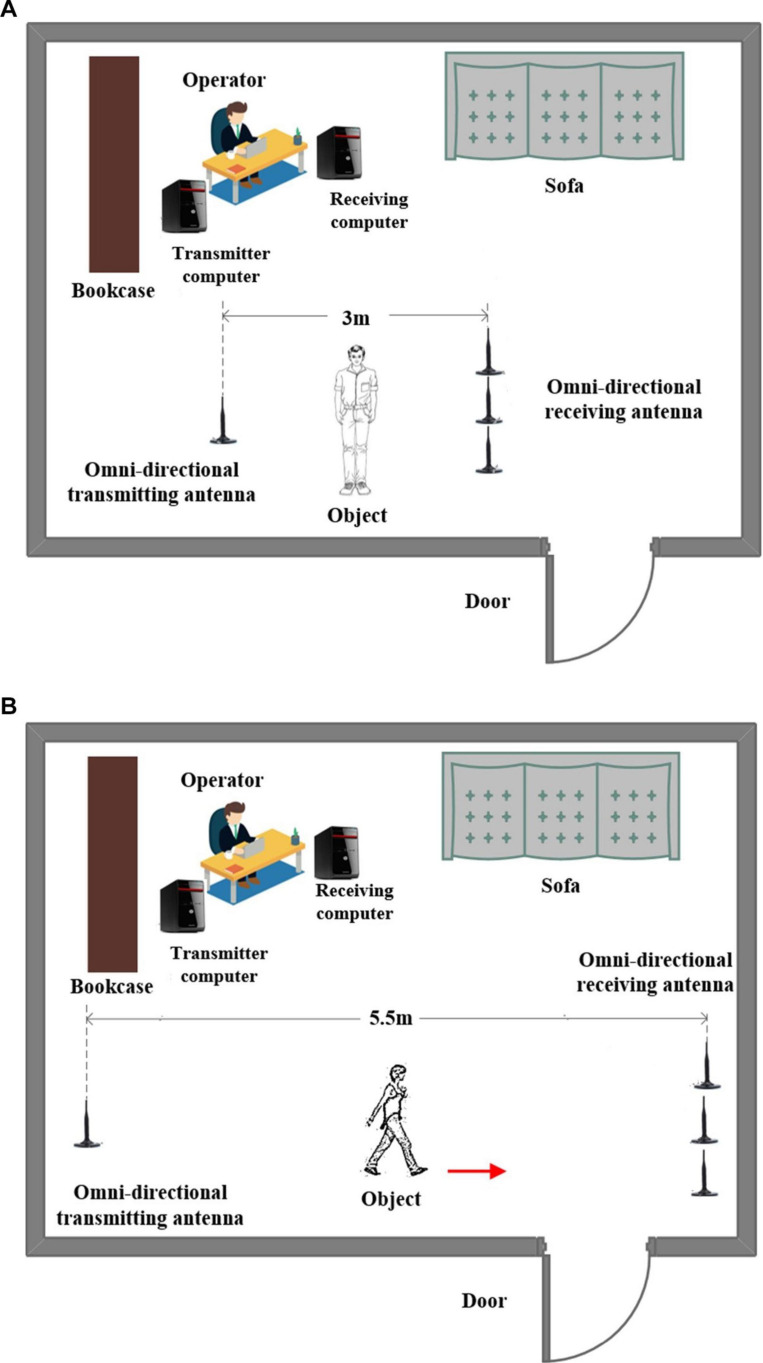
Experiment scene for Romberg’s test and gait measurement. **(A)** Romberg’s test measurement and **(B)** gait measurement.

The experimental equipment includes two industrial control computers equipped with facilities conforming to the IEEE 802.11n standard. The transmitter is equipped with an omnidirectional antenna, and the receiver is equipped with three omnidirectional antennas. Since each antenna receives packets containing 30 subcarriers, we will get 3× 30 subcarriers for each packet at the receiving end, which greatly increases the data size. The experimental scene is shown in Figure 5.

In the experiment, 10 subjects are considered; they are divided into two groups with five people in each group. We set the contract awarding frequency to 200 Hz. For Romberg’s test, we collected a total of 12 s of data, including 6 s for the open eyes stage and 6 s for the close eyes stage. For the gait detection experiment, considering the site constraints and the walking speed between different objects, we collected a total of 5 s of data, the amount of data is enough to distinguish the abnormal gait.

For each subject, Romberg’s test and gait detection were repeated 24 times. We collected three sets of data each day and collected the complete data in about 1 week. There are 120 sets of experimental data for each of the test items for sensory ataxia and cerebellar ataxia. At the same time, we also collected 120 sets of Romberg’s test and gait detection data under normal conditions as a reference.

## The Data Processing

**FIGURE 6:**
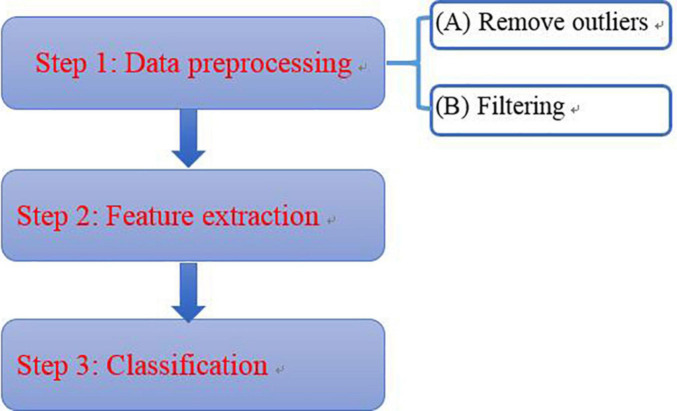
Data processing flow.

Due to the noise in the environment, to ensure the credibility and accuracy of the results, the data is processed considering the following steps (Figure 6):

### Data Preprocessing

#### Remove Outliers

**FIGURE 7:**
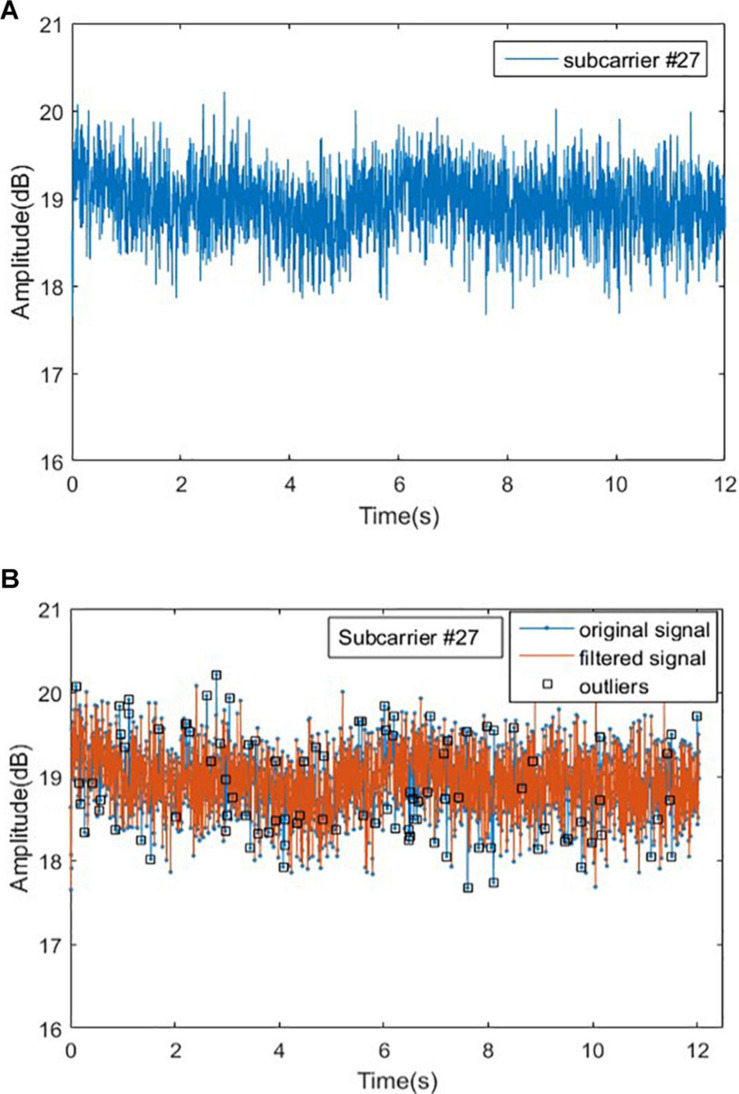
Original signal and outliers for normal people in Romberg’s test. **(A)** Original signal for normal people and **(B)** outliers for normal people.

In order to explain the method of removing outliers, we randomly select a group of original experimental data from normal person in Romberg’s test, and randomly select a subcarrier (No. 27). The signal curve of the subcarrier is shown in Figure 7A. When a normal person performs Romberg’s test, the body shake is within a certain range, and the signal curve of subcarrier should be relatively stable, but in Figure 7B, there are many burrs in the signal curve and the volatility is large. We could also use the Hampel function based on the Pauta criterion to complete the removal of the outliers in the original signal (Li et al., 2016).

#### Signal Denoising

After removing the outliers from the original signal, the noise contained in the original signal will be filtered out. Conventional filters mainly include linear filters and nonlinear filters such as mean filter and Wiener filter. The shortcoming of the traditional denoising method is that the entropy after signal transformation would increase, the non-stationary characteristics of the signal cannot be characterized, and the correlation of the signal cannot be obtained. To overcome these issues, the wavelet transform is used.

**FIGURE 8:**
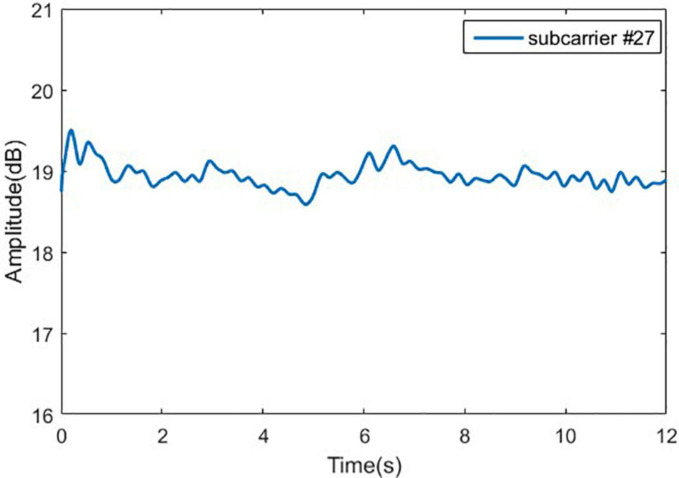
Wavelet transform filtering effect.

Wavelet transform has the characteristics of low entropy, multi-resolution, and flexible selection of wavelet basis functions. In this paper, the wavelet soft threshold method is used for signal denoising, which is simple to implement, and very suitable for processing low SNR (Poornachandra, 2008). We denoise the signal according to the following steps: (1) Wavelet decomposition; (2) Threshold quantization of high-frequency coefficients of wavelet decomposition; and (3) Wavelet reconstruction. The wavelet function selected in this work is sym8, and the signal is decomposed into 5 layers. At the same time, in step 2, the threshold is dynamically adjusted according to the noise level of different decomposition layers. The experimental results show that the wavelet transform has smooth denoising effect, which is shown in Figure 8.

### Feature Extraction

#### Select Subcarrier

**FIGURE 9:**
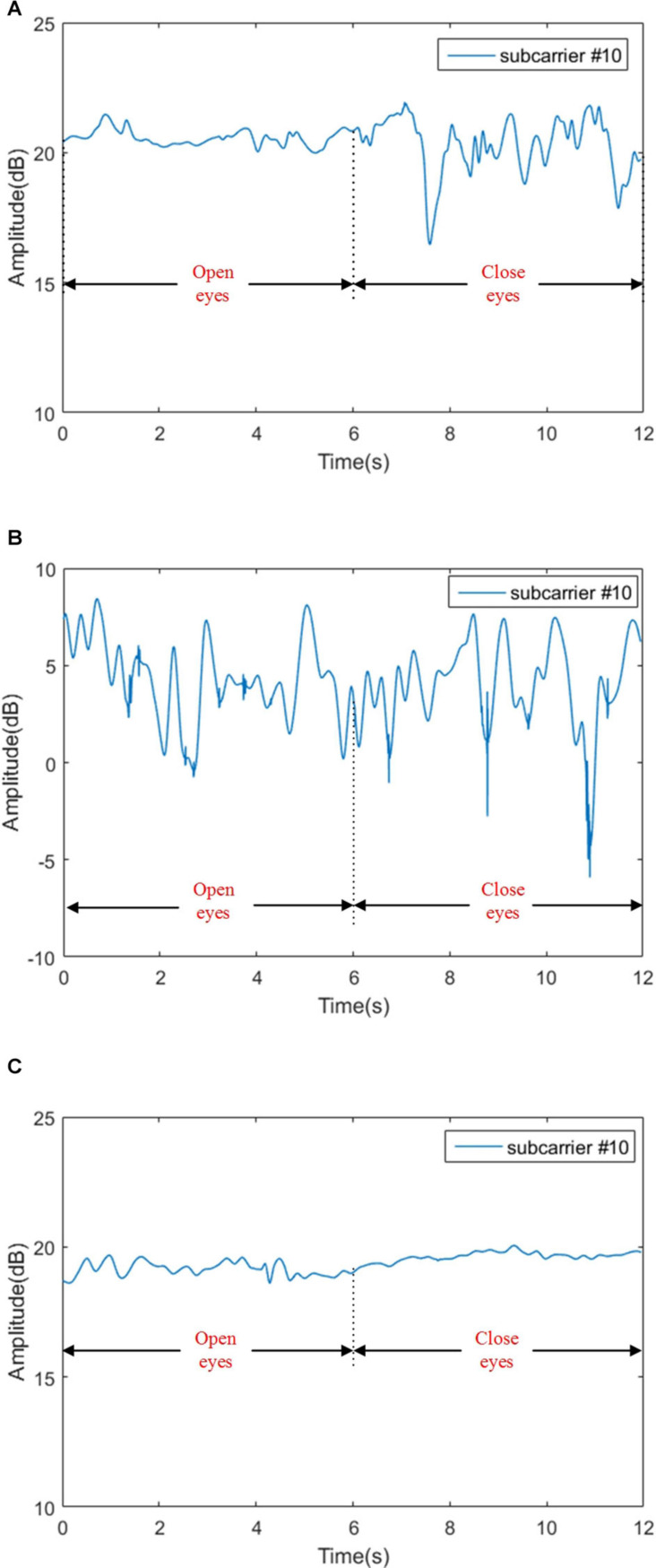
Signal amplitudes for sensory ataxia subject, cerebellar ataxia subject and normal person in Romberg’s test. **(A)** Signal amplitudes for sensory ataxia subject, **(B)** signal amplitudes for cerebellar ataxia subject, and **(C)** signal amplitudes for normal person.

**FIGURE 10:**
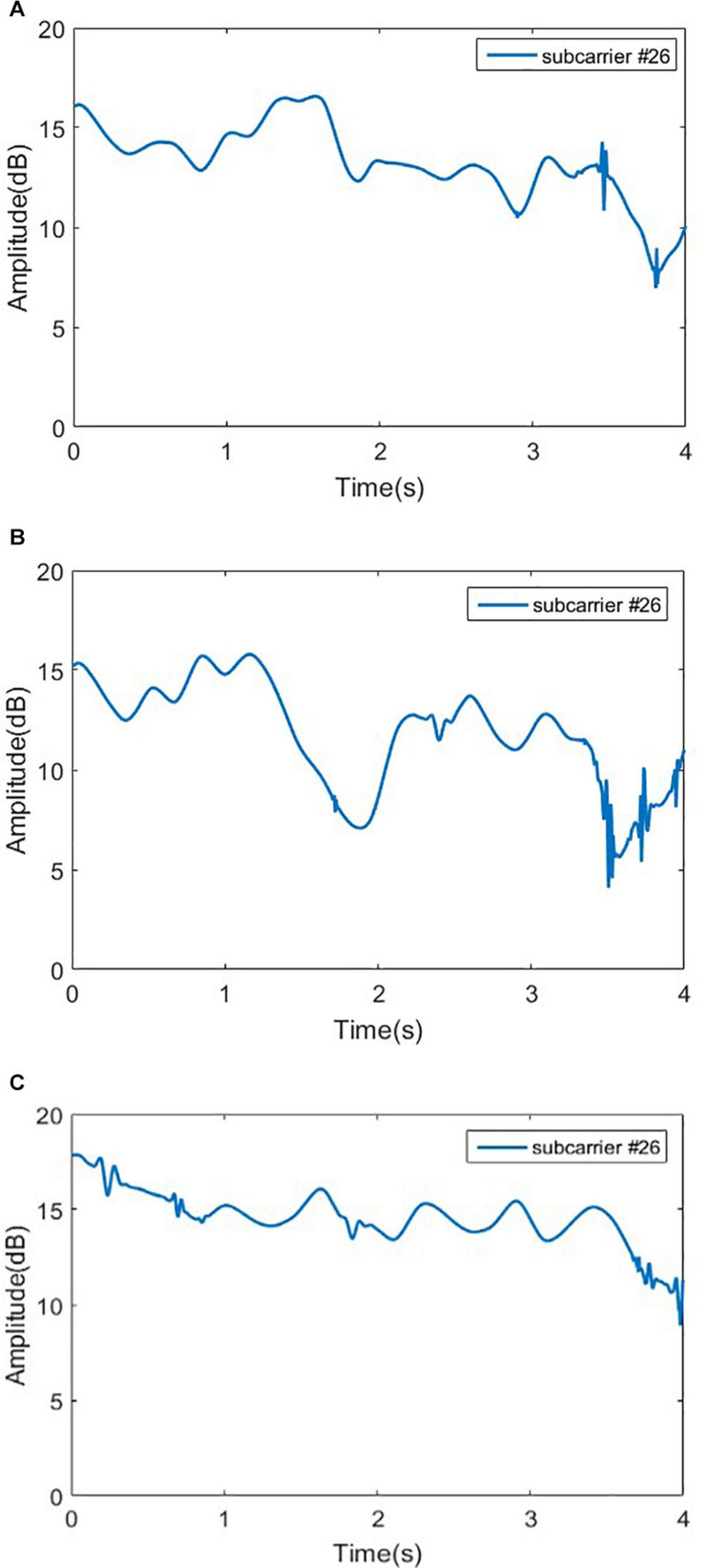
Signal amplitudes for sensory ataxia subject, cerebellar ataxia subject and normal person in gait test. **(A)** Signal amplitudes for sensory ataxia subject, **(B)** signal amplitudes for cerebellar ataxia subject, and **(C)** signal amplitudes for normal person.

Before feature extraction, it is necessary to pick out the appropriate subcarriers. We know that when the variance of a set of data is larger, more information will be contained. According to the principle of maximum variance, for Romberg’s test, we select the 10th subcarrier of the third antenna; and for the gait detection experiment, we select the 26th subcarrier of the second antenna. The experimental data of selected subcarriers are shown in Figures 9, 10, respectively.

#### Extracting Feature of the Romberg’s Test Data

As seen in Figure 9C, in Romberg’s test, the normal person can maintain balance even if he blinks or closes his eyes; slight fluctuations might be caused by the breathing of the object and the noise in the environment. Patients with sensory ataxia can maintain body balance during the blinking phase, and the body violently shakes during the closed eyes stage, resulting in a waveform that is basically stable in the blinking phase and unstable in the closed eyes phase, as shown in Figure 9A. For patients with cerebellar ataxia, whether they are blinking or closing their eyes, the body is shaking sharply, and the waveform fluctuates sharply, as shown in Figure 9B.

Since different groups in the Romberg’s test have different time domain waveforms, in order to improve the efficiency of the classification model training, only the time domain characteristics are extracted and are shown in Table 2.

**TABLE 2:** The extracted time domain features of the Romberg’s test data.

**Features**	**Calculation formula**
Mean value	$Y_{M\,V}=\frac{1}{N}\,\sum_{i=1}^{N}x_{i}$
Standard deviation	$Y_{S\,D}=\sqrt[{2}]{\frac{1}{N-1}\,\sum_{i=1}^{N}{\left(x_{i}-Y_{M\,v}\right)}^{2}}$
Root mean square	$Y_{R\,M\,S}=\sqrt[{2}]{\frac{1}{N}\,\sum_{i=1}^{N}x_{i}^{2}}$
Peak to peak value	*Y*_*PPV*_ = *max*(*x*_*i*_)−*min*(*x*_*i*_)(*i* = 1,2,…,*N*)
Kurtosis	$Y_{K}=\frac{\frac{1}{N}\,\sum_{i=1}^{N}{\left(\left|x_{i}\right|-Y_{M\,V}\right)}^{4}}{Y_{R\,M\,S}{}^{4}}$
Skewness	$Y_{S}=\frac{\frac{1}{N}\,\sum_{i=1}^{N}{\left(\left|x_{i}\right|-Y_{M\,V}\right)}^{3}}{Y_{R\,M\,S}{}^{3}}$
Peak factor	$Y_{P}=\frac{\max\left(x_{i}\right)}{Y_{R\,M\,S}}\left(i=1,2,\ldots ,N\right)$
Waveform factor	$Y_{W}=\frac{N*Y_{R\,M\,S}}{\sum_{i=1}^{N}\left|x_{i}\right|}\left(i=1,2,\ldots ,N\right)$

The physical significance of each time domain feature is as follows: the mean value describes the stable component of the signal, the mean square value reflects the energy of the signal, the standard deviation can represent the degree of dispersion between the signal sampling points, the kurtosis reflects the impact characteristics in the signal, and the skewness reflects the asymmetry of the signal. The peak-to-peak value reflects the signal amplitude range. The peak factor can be used to detect whether there is an impact in the signal. The physical meaning of the waveform factor in the electronic field can be understood as the ratio of the DC signal of the same power to the original AC signal, and its value is greater than or equal to 1.

#### Extracting Feature of the Gait Detection Data

**FIGURE 11:**
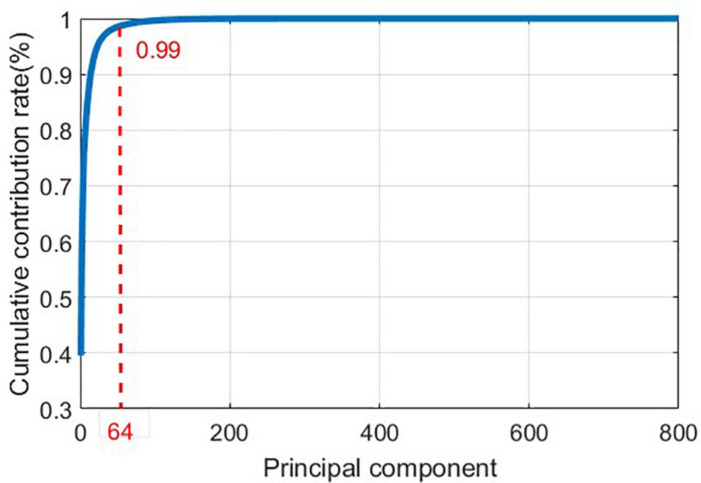
Principal component cumulative contribution rate curve.

As it can be seen in Figure 10, the time domain waveforms of the three gaits have little discrimination. To ensure the accuracy of the results, Principal Component Analysis (PCA) (Wold et al., 1987) is adopted to reduce the dimensionality of the original data, and the cumulative contribution rate of each principal component is shown in Figure 11.

In order to avoid information loss in the original data and to eliminate redundant information, the first 64 principal components are extracted as features.

### Classification

After these steps, we have obtained the dataset of Romberg’s test and gait detection. Each dataset contains 360 samples, including normal, sensory ataxia and cerebellar ataxia. To increase the credibility and accuracy of the results, we adopted a four-fold cross-validation (Demsar, 2006) method to divide the training set and test set, and adopted three classification algorithms including BP Neural Network, SVM, and RF.

## Experimental Results and Discussion

### Experimental Results

**FIGURE 12:**
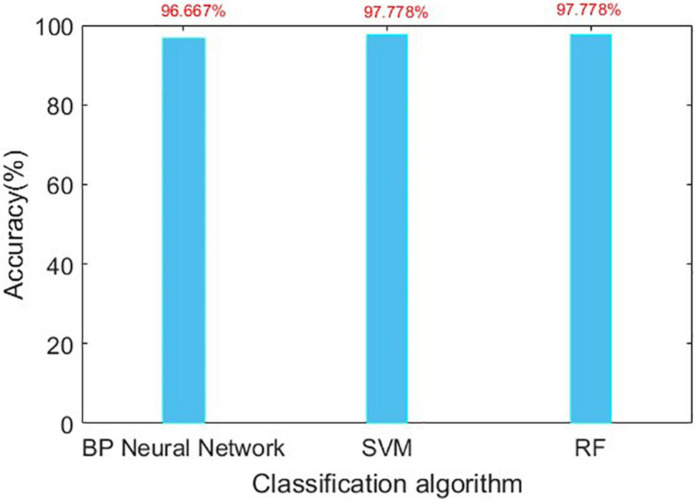
Accuracy of three algorithms in Romberg’s test.

**FIGURE 13:**
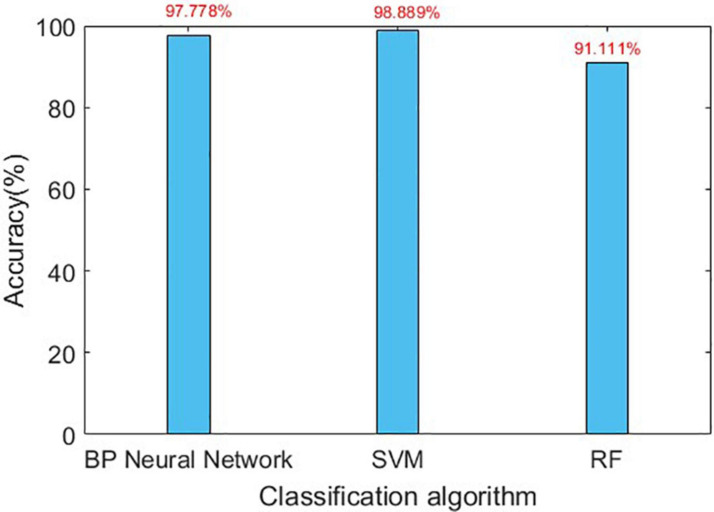
Accuracy of three algorithms in gait detection.

The confusion matrix for the results are shown in Tables 3, 4, and the accuracies of each algorithm is shown in Figures 12, 13.

**TABLE 3:** Confusion matrix for Romberg’s test.

**Classification algorithm**	Actual type (Each test set contains 3*30 samples)	**Predict type (Number of sample)**		
		**Normal**	**Sensory ataxia**	**Cerebellar ataxia**
BP Neural Network	Normal	29	1	0
	Sensory ataxia	2	28	0
	Cerebellar ataxia	0	0	30
SVM	Normal	29	1	0
	Sensory ataxia	1	29	0
	Cerebellar ataxia	0	0	30
RF	Normal	29	1	0
	Sensory ataxia	1	29	0
	Cerebellar ataxia	0	0	30

**TABLE 4:** Confusion matrix for gait detection.

**Classification algorithm**	Actual type (Each test set contains 3*30 samples)	**Predict type (Number of sample)**		
		**Normal**	**Sensory ataxia**	**Cerebellar ataxia**
BP Neural Network	Normal	30	0	0
	Sensory ataxia	0	28	2
	Cerebellar ataxia	0	0	30
SVM	Normal	29	0	1
	Sensory ataxia	0	30	0
	Cerebellar ataxia	0	0	30
RF	Normal	24	1	5
	Sensory ataxia	0	30	0
	Cerebellar ataxia	0	2	28

### Discussion

It can be seen from Figures 12, 13 that for Romberg’s test, only the time domain features are extracted, and all the three algorithms can achieve an accuracy of more than 96%; for gait detection, PCA is used for dimensionality reduction; the accuracies of BP Neural Network and SVM algorithm are above 97%. From Table 4, the source of error rate of RF algorithm is mainly used to identify the normal person between sensory ataxia and cerebellar ataxia; for gait recognition, BP Neural Network and SVM are considered; for Romberg’s test, all three classification algorithms are suitable.

From Table 3, we can see that in Romberg’s test, very high precision is achieved, and the error rate is mainly due to the misjudgment of normal and sensory ataxia. The reason for this is that in Romberg’s test, cerebellar ataxia subjects are not stable whether they open or close their eyes, while sensory ataxia subjects and normal subjects remained stable during eye opening; the only difference between the two is that sensory ataxia subjects shake after closing their eyes, and closing eyes have no effect on normal people. The time domain waveform from Figure 9 can also give the corresponding conclusion. The ability to maintain body balance is related to the age, gender and the length of time for standing of the individual. Normal people may have slight shaking in the Romberg’s test, symptoms of patients with sensory ataxia may be mild, which may cause confusion. It is worth mentioning that it’s difficult to distinguish normal subjects from sensory ataxia subjects completely in Romberg’s test, but cerebellar ataxia can be detected. In order to ensure the experimental results more reliable, gait detection experiments are also performed, and the two experiments confirmed reliability of the system.

## Conclusion

Sensory Ataxia and Cerebellar Ataxia are neurological diseases which affect the patients’ quality of life seriously; therefore, their detection at early stage are very important and necessary. In this paper, non-contact wireless sensing technology has been proposed to discriminate symptoms between the two diseases. The advantages include improvement of comfort, overcoming self-consciousness enhancing, etc. The main merit of the system lies in its convenience and price cost advantage. We firstly preprocess the data by removing outliers, wavelet transform filtering, then data features are extracted, finally, we use BP Neural Network, SVM, RF machine learning algorithms to train the model. The experimental results show that most of the algorithms can achieve more than 96% prediction accuracy, which can effectively discriminate between sensory ataxia and cerebellar ataxia, and prove that the technical scheme described in this paper is effective. Next, we will further explore the application of C-Band wireless sensing technology in healthcare, and propose more clinical application programs to make clinical detection more accurate, reliable and smarter, so as to reduce the burden on clinicians and patients.

## Data Availability Statement

The raw data supporting the conclusions of this article will be made available by the authors, without undue reservation.

## Ethics Statement

The studies involving human participants were reviewed and approved by the Northwest Women’s and Children’s Hospital, Xi’an Jiaotong University Health Science Center. The patients/participants provided their written informed consent to participate in this study.

## Author Contributions

QZ: manuscript writing. XZ, YL, and XY: guidance. XY: editing, project management, and funding. QA: verification. All authors contributed to the article and approved the submitted version.

## Conflict of Interest

The authors declare that the research was conducted in the absence of any commercial or financial relationships that could be construed as a potential conflict of interest.
